# Developing Empirical Decision Points to Improve the Timing of Adaptive Digital Health Physical Activity Interventions in Youth: Survival Analysis

**DOI:** 10.2196/17450

**Published:** 2020-06-10

**Authors:** Adrian Ortega, Christopher C Cushing

**Affiliations:** 1 Clinical Child Psychology Program University of Kansas Lawrence, KS United States; 2 Schiefelbusch Life Span Institute University of Kansas Lawrence, KS United States

**Keywords:** telemedicine, exercise, physical activity, adolescent

## Abstract

**Background:**

Current digital health interventions primarily use interventionist-defined rules to guide the timing of intervention delivery. As new temporally dense data sets become available, it is possible to make decisions about the intervention timing empirically.

**Objective:**

This study aimed to explore the timing of physical activity among youth to inform decision points (eg, timing of support) for future digital physical activity interventions.

**Methods:**

This study comprised 113 adolescents aged between 13 and 18 years (mean age 14.64, SD 1.48 years) who wore an accelerometer for 20 days. Multilevel survival analyses were used to estimate the most likely time of day (via odds ratios and hazard probabilities) when adolescents accumulated their average physical activity. The interacting effects of physical activity timing and moderating variables were calculated by entering predictors, such as gender, sports participation, and school day, into the model as main effects and tested for interactions with the time of day to determine conditional main effects of these predictors.

**Results:**

On average, the likelihood that a participant would accumulate a typical amount of moderate-to-vigorous physical activity increased and peaked between 6 PM and 8 PM before decreasing sharply after 9 PM. Hazard and survival probabilities suggest that optimal decision points for digital physical activity programs could occur between 5 PM and 8 PM.

**Conclusions:**

Overall, the findings of this study support the idea that the timing of physical activity can be empirically identified and that these markers may be useful as intervention triggers.

## Introduction

### Background

Less than 25% of the youth attain the recommended amounts (60 min per day) of moderate-to-vigorous physical activity (MVPA) [1]. Despite the importance of physical activity for health [2-4], it is well documented that rates of daily MVPA decrease from childhood to adolescence about 40 min each year between ages 9 and 15 years [5]. Accordingly, adolescence is a stage when the youth are likely to exhibit declines in physical activity [6], and therefore, empirical research investigating the patterns of physical activity in adolescence may have value for future interventions to forestall this decline.

The daily activity of youths is in four levels: sleep, sedentary activity, light activity, and MVPA [7,8]. The time spent in each of these levels is strongly linked, such that reducing or increasing the time spent in one of these levels is inversely related to how much time is spent in other levels [9,10]. As the youth choose to pursue sedentary activities (eg, watching television), the time and *opportunity* available for exercise decrease. In other words, there is a finite number of minutes in the day, and every passing minute of physical inactivity results in a loss of opportunity for physical activity.

There is growing interest among behavioral scientists to translate efficacious behavioral interventions for improving physical activity into digital modalities [11,12]. Although mobile health interventions for improving health behavior are efficacious, these interventions primarily use technologies to send reminder messages to users [13]. Moreover, high *nonusage* attrition and declined user engagement throughout the intervention are concerns in the digital health literature [14,15]. Greater intervention precision, such as timely support, may improve digital intervention effectiveness and engagement [16].

Just-in-time adaptive interventions (JITAIs) capitalize on advanced digital technologies and computer automation to tailor digital interventions to the user’s needs and deliver these interventions at opportune moments [16]. A critical component of JITAIs is the decision point or timing of support [16]. JITAIs seek to intervene at critical windows of opportunity for each user to maximize effectiveness and engagement while also minimizing waste or participant burden; however, consistent methods of developing decision points have not been established [16]. In the absence of a clear theory to guide when JITAIs should intervene, it is valuable to empirically identify critical windows of opportunity that can be adjusted based on users’ needs rather than at moments made *a priori* by investigators as intervention decision points [17]. However, very few digital physical activity interventions have adjusted the timing of intervention delivery based on observed patterns of physical activity [17]. A recent meta-analysis [18] revealed that most physical activity JITAIs modified decision points based on rational decisions, such as interventionist-defined [19-21] or user-defined times [22]. Physical activity JITAIs dynamically tailored their interventions at the individual level based on context-aware sensing and machine learning algorithms [23,24], how and the extent to which the decision points were modified beyond content adaptations, and its unique effect on changes in physical activity remains unclear. In other words, despite these studies, a research gap still remains regarding when adolescents allocate time to physical activity and how we can best leverage these times to improve exercise attainment digitally.

An investigation of the temporal windows of users’ previous exercise patterns can help pinpoint subsequent decision points for intervention. It may prove beneficial to investigate the timing of activity in the youth to identify critical windows of opportunity for when users allocate time to engage in physical activity. Thus, digital interventions can optimize intervention delivery and maximize engagement in physical activity when it is likely to occur for users. By delivering support at the empirically observed moments and at random, interventionist-defined users, or user-selected decision points, may be more likely to exhibit health-promoting behaviors, such as physical activity.

### Study Aims and Hypotheses

The primary objective of this study (aim 1) was to explore the time of day when an adolescent accumulated their average physical activity. This aim explored the most likely hour by which an adolescent would have accumulated their average physical activity, *given that it had not occurred already*. As adolescents are unlikely to meet the recommended 60 min of MVPA each day, this study examined the likelihood that each participant met their average unique MVPA. Aim 1 was exploratory, with no *a priori* hypothesis postulated.

The secondary objective (aim 2) was to explore day-level and individual-level factors that moderate the likelihood of accumulating physical activity at the time of day found in the primary aim of this study. For this study, the determinants selected for moderation analyses included empirically derived variables previously shown to be correlated with physical activity, such as gender, BMI, sports participation, and school day [25-30]. The primary focus of aim 2 was to determine the magnitude of group differences in the odds of meeting their average MVPA. Gender and BMI are related to exercise attainment and could influence when adolescents meet their average MVPA. For example, because males and individuals with lower BMI engage in more physical activity, they may be more likely to meet their average MVPA compared with females or individuals with a higher BMI. In addition, participation in organized sports or days in which a person attends school might also permit or constrain opportunities to exercise, thereby affecting timing as well. It is hypothesized that youths who participate in sports will obtain higher odds ratios (ORs) of meeting their average MVPA compared with youths who do not participate in sports and that youths will obtain higher ORs of meeting their average MVPA on days in which they attended school than on days in which they did not.

Finally, the third aim (aim 3) was to generate decision points to improve the timing of digital physical activity interventions, given the results from aims 1 and 2. Sets of decision points were inferred from hazard and survival probabilities (see Statistical Analyses section). Decision points were generated for moderators only if the following two conditions were satisfied: (1) if a moderator was found to be significantly different between groups of individuals and (2) if the differences between these groups were as hypothesized in aim 2. These criteria were set forth so that decision points would only be made for subgroups in which there were meaningful differences in the timing of physical activity and to eliminate ineffectual moderators.

## Methods

### Recruitment

Participants were recruited as part of a study examining the associations between various psychological constructs and physical activity behaviors. Participants learned about the study through flyers posted around the local community. Interested participants were instructed to contact study personnel via phone calls to screen for eligibility. Participants enrolled in the study were aged between 13 and 18 years and lived at home with their caregiver or caregivers. Participants were excluded if they had any significant physical maladies that would limit physical activity, visual impairments, or an inability to read at their corresponding grade level. These exclusion criteria were in place to ensure a valid assessment of physical activity. A total of 121 adolescents were recruited to participate.

### Procedures

At the baseline visit, participants reviewed the study information and an institutional review board–approved informed consent form with the research staff and gave consent to participate. For participants aged <18 years, their parents provided informed consent, and adolescents provided informed assent. The participants then completed a demographics questionnaire, a planned activity calendar, and were oriented to the accelerometer. Participants were instructed to wear the accelerometer on their nondominant wrist for the entire 20-day study period [31]. Finally, the research staff measured each participant’s height and weight. Adolescents then wore the accelerometer for 20 days. At the end of the 20 days, participants returned for a laboratory exit visit to return the accelerometers. When all procedures were completed, participants received up to US $40 financial compensation for both laboratory visits and completing the surveys (US $25) and for wearing the accelerometer for at least 18 of the required 20 days (US $15). Data were collected year-round, beginning in June 2015 and ending in December 2017, across multiple seasons, and when the youth were in and out of school (ie, weekends and weekdays as well as during summer/winter breaks).

### Ethics Approval and Consent to Participate

In accordance with the revised Common Rule, this study was approved by the sponsoring institution’s human subjects’ protection committee, and all participants provided informed consent and assent.

### Sample Characteristics

Participants were excluded from the current analyses if they had less than 4 days of valid accelerometer wear (eg, ≥8 hours of accelerometer wear time per day, n=8). Participants in the current analyses (N=113) were aged between 13 and 18 years (mean 14.64, SD 1.48 years). The sample was 37.2% men (32/113) and 62.8% women (71/113). In terms of caregiver demographics, 66.4% (75/113) of parents were married; 22.1% (25/113) were divorced, separated, or widowed; and 11.5% (13/113) were never married. Moreover, 61.1% (69/113) of mothers and 63.7% (72/113) of fathers attained a college education or higher. The majority of the sample (61.1%, 69/113) reported an approximate family income greater than US $60,000. The sample was 78.8% (89/113) Caucasian, 8.0% (9/113) Latino/Latina, 4.4% (5/113) African American, 1.8% (2/113) multiracial, 0.9% (1/113) Native American, and 2.7% (3/113) other.

### Measures and Measurement

#### Demographics

Adolescents self-reported basic demographic information, including date of birth, age, sex, race, level(s) of parental education, and approximate family income.

#### Physical Activity

The ActiGraph wGT3X-BT accelerometer (ActiGraph LLC) objectively measured participants’ MVPA throughout the duration of the 20-day study period. The accelerometers were programmed to sample movements at a rate of 30 Hz on three different axes, as in previous studies [32] measuring physical activity in the youth. Irrelevant activity periods such as sleep periods and nonwear periods were identified using the Sadeh algorithm and the Troiano algorithm, respectively, and thus removed from the daily activity counts [33,34]. Only days when participants wore the accelerometer for 8 hours or more were included in this study. As in previous studies using wrist-worn accelerometers in youths, the Chandler algorithm was used to identify total minutes of MVPA per day [32,35]. Given previous research demonstrating participant reactivity to accelerometer measurement during the first days of the study, research personnel removed the first 3 days of accelerometer activity data for each participant because of potential activity-based reactivity [36]. After removing these days, the mean of each participant’s unique MVPA was created by calculating the mean of their daily MVPA attainment.

#### BMI

BMI was calculated using the Center for Disease Control and Prevention (CDC) growth charts [37]. Participants’ height in centimeters, weight in kilograms, sex, and age in months at the time of study initiation were used to compute BMI. BMI was calculated using the SAS program for the CDC growth charts found on the CDC website [38].

#### Sports Participation

During the baseline session, participants were asked if they had any planned physical activity, such as involvement in organized sports, for the next 20 days. Similar to previous studies, participants were dichotomously categorized into 2 groups: involved in organized sports and uninvolved in organized sports based on their self-reported activity involvement [25,39-41].

#### School Day

Research personnel classified daily activity patterns into categorical variables: school day and nonschool day to better capture the variability among physical activity patterns for days when youths are in school or not. As in previous retrospective studies investigating the magnitude of physical activity and sedentary time differences when youths are in and out of school, research personnel used the school calendar to classify school days vs nonschool days [42].

### Statistical Analyses

To address the aims of this study, multilevel survival analyses using logistic regression were conducted to examine the hour of day when adolescents accumulated their average physical activity. Predictors were entered into the model as main effects and subsequently tested as interactions with the time of day to determine the conditional main effects of study moderators on the time of day when adolescents accumulated their average physical activity. For the purposes of this project, the time of day was analyzed as a discrete time variable. Using a special case of logistic regression, a hazard function, or the probability of the event occurring before the time (hour of day, in this case), conditional on no earlier occurrence, was estimated [43].

To estimate hazard functions, multiple smoothing procedures using polynomial functions of time were tested [43]. For each survival analysis, polynomials of time were entered into the model as linear, quadratic, and cubic predictors of the event. Each survival analysis was estimated using maximum likelihood estimation based on Laplace approximation in SAS PROC GLIMMIX. Nested model comparisons using the chi-square difference of the estimated −2×Log likelihood ratio tests for each of these models were evaluated to determine the best fitting model for the polynomial effect of time. Predictors as well as interactions between predictors and time of day were then entered into the hazard model. Each of these hazard models was estimated separately. In the event of a significant interaction of a predictor and time of day, the effect of these interactions was determined by comparing the OR of the estimates by summing all parameter estimates multiplied by their respective variables and then using the inverse link function (ie, *e*^log (y=1)^) to translate estimated logits into ORs. To compare the estimates, ORs were calculated by inserting meaningful values into the explanatory predictors in the regression equations. To address aims 1 and 2 OR estimates were used to determine the most likely hour of average MVPA accumulation and group differences in the timing of MVPA obtainment using 8 AM as the reference hour. To investigate whether there were significant differences in the magnitude of the ORs for the time of average MVPA accumulation between groups at *P* values <.05 level of significance, the 95% CIs around the estimated ORs were compared. OR estimates that did not overlap based on their given 95% confidence bands were considered statistically significant at the *P* value <.05 level [44]. For each moderator, statistical differences between groups at every hour of the day were examined.

If the ORs were statistically significant for a window of time in the hypothesized direction, decision points using hazard and survival probabilities were developed, as stated in aim 3. The hazard and survival probabilities were created by translating the estimated logits into probabilities. Survival probabilities represent the cumulative risk that an individual would not have met the event at a certain time. To estimate the survival probability at each hour, we multiplied the complement of the hazard probabilities (ie, 1 × probability) for that hour and all previous hours [45].

## Results

### Preliminary Analyses

On average, participants accumulated 30.91 min (SD 30.94; range 0-98.16) of MVPA per day and, therefore, did not meet the recommended guidelines. Participants wore the accelerometer for an average of 17.60 (SD 4.5; range 1-25) valid wear days.

### Aim 1

**Figure 1:**
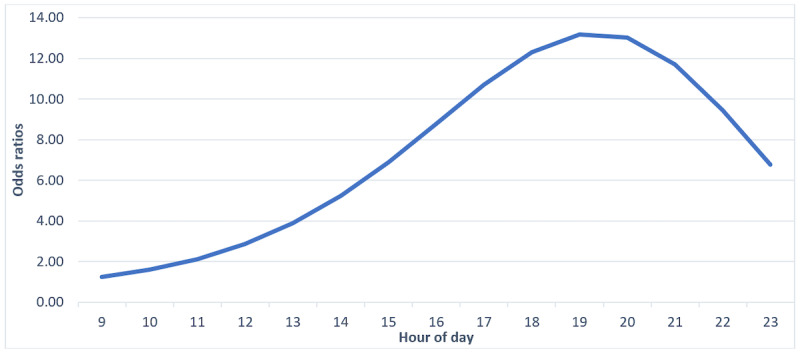
Odds ratios of obtaining average moderate-to-vigorous physical activity before each hour of day.

After inserting sequential polynomials of time, nested model comparisons indicated that a cubic function of time was the best fitting model. On average, the likelihood that a participant would accumulate their own average MVPA increased and peaked between 6 PM and 8 PM (OR 13.19-13.02) before decreasing sharply after 9 PM (Table 1 and Figure 1). No participants met their average MVPA before 8 AM or after 11 PM. For this reason, tables and figures displaying results for aims 1 to 3 reflect the ORs, hazard probabilities, and survival probabilities that an individual met their average MVPA by that hour, conditional on no earlier occurrence, beginning between the hours before 8 AM and terminating before 11 PM.

**Table 1:** Odds ratio estimates of obtaining average moderate-to-vigorous physical activity before hour of day.

Hour	Frequency^a^	Odds ratio estimates (95% CI)
Before 8 AM (reference)	0	N/A^b^
Before 9 AM	5	1.24 (1.31-1.36)
Before 10 AM	21	1.60 (1.34-1.91)
Before 11 AM	18	2.13 (1.67-2.72)
Before noon	24	2.87 (2.12-3.7)
Before 1 PM	22	3.90 (2.73-5.56)
Before 2 PM	24	5.24 (3.53-7.77)
Before 3 PM	50	6.90 (4.51-10.55)
Before 4 PM	55	8.79 (5.63-13.72)
Before 5 PM	85	10.70 (6.78-16.89)
Before 6 PM	89	12.30 (7.77-19.45)
Before 7 PM	82	13.19 (8.38-20.73)
Before 8 PM	91	13.02 (8.38-20.22)
Before 9 PM	79	11.70 (7.66-17.84)
Before 10 PM	43	9.45 (6.3-14.12)
Before 11 PM	32	6.77 (4.6-9.94)

^a^Frequency represents the number of instances in which users met their average moderate-to-vigorous physical activity before hour of day.

^b^N/A: not applicable.

### Aim 2

**Figure 2:**
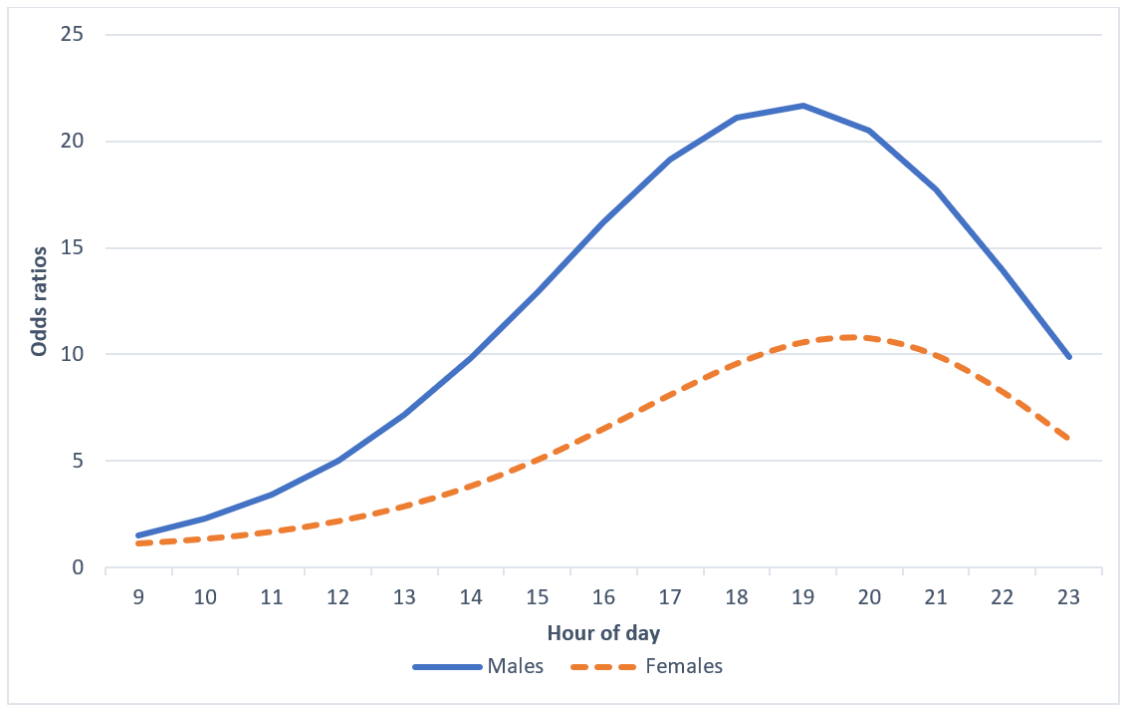
Sex differences in odds ratios of obtaining average moderate-to-vigorous physical activity before each hour of day.

Compared with 8 AM, male adolescents had significantly higher odds of obtaining their average MVPA only between the 8 AM and noon window compared with female adolescents (Table 2 and Figure 2).

**Figure 3:**
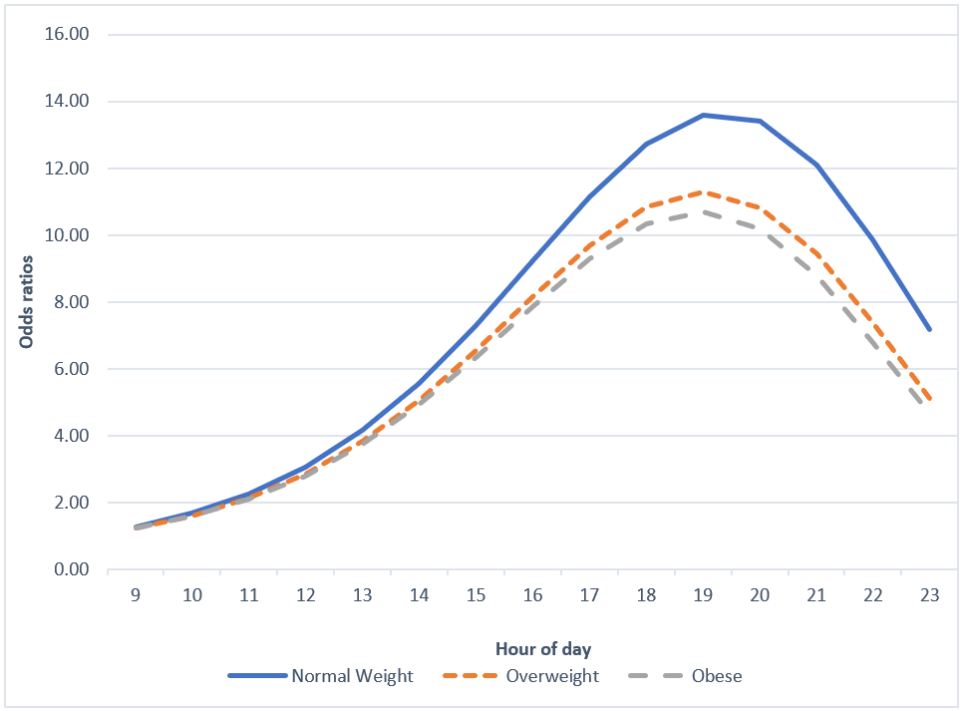
BMI differences in odds ratios of obtaining average moderate-to-vigorous physical activity before each hour of day.

BMI did not significantly moderate the relationship between time of day and MVPA attainment (Table 3 and Figure 3).

**Figure 4:**
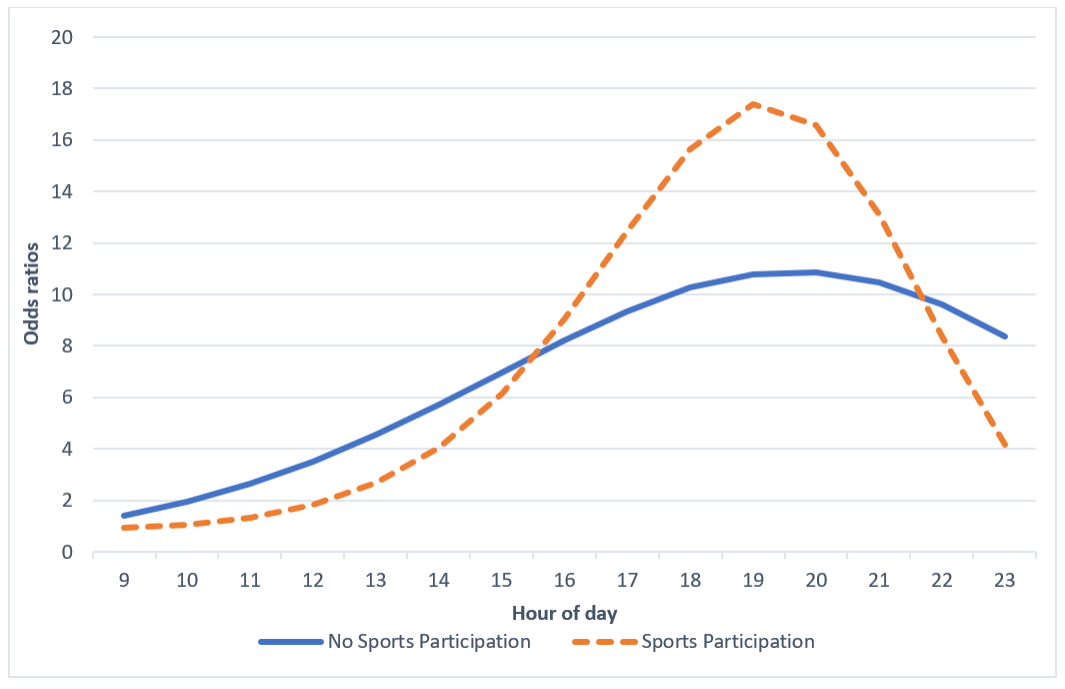
Involvement in organized sports differences in odds ratios of obtaining average moderate-to-vigorous physical activity before each hour of day.

In addition, adolescents involved in organized sports had significantly lower odds of attaining their average MVPA between 8 AM and 10 AM (Table 4 and Figure 4).

**Figure 5:**
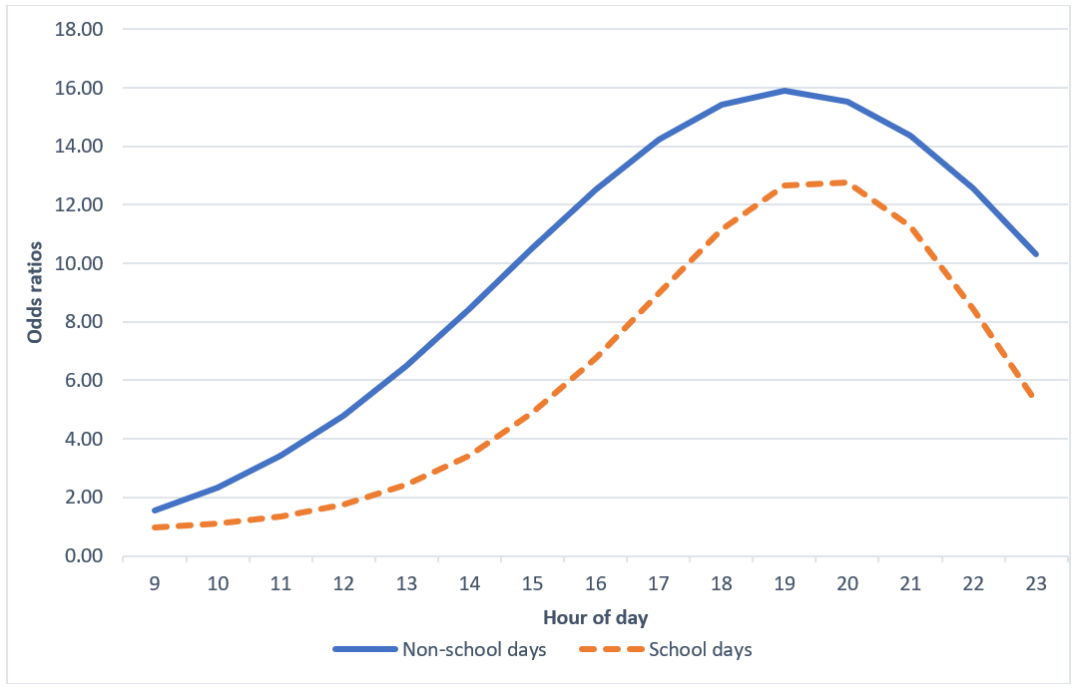
School day differences in odds ratios of obtaining average moderate-to-vigorous physical activity before each hour of day.

Adolescents had significantly higher odds between the 8 AM and noon window, compared with 8 AM, of meeting their average MVPA on nonschool days compared with school days (Table 5 and Figure 5).

**Table 2:** Moderating effect of sex on odds ratio estimates of time of average moderate-to-vigorous physical activity accumulation.

Hour	Odds ratio estimates (95% CI)	
	Males^a^ (n=42)	Females^b^ (n=71)
Before 8 AM (reference)	N/A^c^	N/A
Before 9 AM	1.52 (1.29-1.79)^d^	1.12 (1-1.25)
Before 10 AM	2.30 (1.8-3.34)^d^	1.33 (1.08-1.64)
Before 11 AM	3.43 (2.42-5.78)^d^	1.66 (1.24-2.23)
Before noon	5.02 (3.21-9.54)^d^	2.16 (1.49-3.11)
Before 1 PM	7.15 (4.2-14.95)	2.86 (1.85-4.38)
Before 2 PM	9.83 (4.87-16.66)	3.81 (2.35-6.11)
Before 3 PM	12.94 (6.07-27.23)	5.04 (3-8.36)
Before 4 PM	16.20 (7.31-35.35)	6.52 (3.76-11.04)
Before 5 PM	19.12 (8.43-42.52)	8.11 (4.61-13.88)
Before 6 PM	21.13 (9.26-47.12)	9.58 (5.41-16.38)
Before 7 PM	21.69 (9.57-47.79)	10.58 (6-19.91)
Before 8 PM	20.52 (9.24-44.12)	10.77 (6.18-17)
Before 9 PM	17.76 (8.23-36.87)	9.96 (5.81-16.15)
Before 10 PM	13.96 (6.7-27.81)	8.24 (4.89-13.01)
Before 11 PM	9.88 (4.9-18.91)	6.02 (3.62-9.27)

^a^Average moderate-to-vigorous physical activity for men=31.48 min.

^b^Average moderate-to-vigorous physical activity for women=30.66 min.

^c^N/A: not applicable.

^d^Significant differences in odds ratios between groups based on nonoverlapping CIs.

**Table 3:** Moderating effect of BMI on odds ratio estimates of time of average moderate-to-vigorous physical activity accumulation.

Hour	Odds ratio estimates (95% CI)		
	Normal weight status^a^ (n=81)	Overweight status^b^ (n=13)	Obese status^c^ (n=19)
Before 8 AM (reference)	N/A^d^	N/A	N/A
Before 9 AM	1.28 (1.16-1.39)	1.25 (1.14-1.36)	1.24 (1.24-1.36)
Before 10 AM	1.68 (1.4-1.97)	1.61 (1.36-1.9)	1.59 (1.34-1.89)
Before 11 AM	2.26 (1.76-2.83)	2.14 (1.68-2.7)	2.10 (1.64-2.68)
Before noon	3.07 (2.25-4.07)	2.86 (2.12-3.83)	2.8 (2.06-3.8)
Before 1 PM	4.16 (2.91-5.81)	3.83 (2.7-5.4)	3.74 (2.61-5.33)
Before 2 PM	5.57 (3.76-8.12)	5.06 (3.42-7.44)	4.93 (3.3-7.35)
Before 3 PM	7.29 (4.79-10.99)	6.54 (4.29-9.92)	6.34 (4.09-9.8)
Before 4 PM	9.22 (5.95-14.24)	8.15 (5.23-12.67)	7.87 (4.95-12.5)
Before 5 PM	11.14 (7.17-17.48)	9.68 (6.13-15.29)	9.30 (5.73-15.09)
Before 6 PM	12.73 (8.11-20.11)	10.83 (6.82-17.27)	10.35 (6.3-17.03)
Before 7 PM	13.58 (8.7-21.44)	11.29 (7.11-18.04)	10.71 (6.48-17.79)
Before 8 PM	13.40 (8.66-20.99)	10.82 (6.85-17.25)	10.18 (6.14-17.01)
Before 9 PM	12.09 (7.91-18.67)	9.43 (6.01-14.95)	8.79 (5.29-14.74)
Before 10 PM	9.86 (6.53-14.96)	7.39 (4.74-11.64)	6.80 (4.09-11.47)
Before 11 PM	7.19 (4.79-10.72)	5.13 (3.31-8.07)	4.66 (2.79-7.94)

^a^Mean moderate-to-vigorous physical activity (MVPA) for normal weight status=31.23 min.

^a^Mean MVPA for overweight status=27.33 min.

^c^Mean MVPA for obese status=32.32.

^d^N/A: not applicable.

**Table 4:** Moderating effect of involvement in organized sports (sports participation) on odds ratio estimates of time of average moderate-to-vigorous physical activity accumulation.

Hour	Odds ratio estimates (95% CI)	
	No sports participation^a^ (n=68)	Sports participation^b^ (n=45)
Before 8 AM (reference)	N/A^c^	N/A
Before 9 AM	1.41 (1.26-1.58)^d^	0.95 (0.81-1.12)
Before 10 AM	1.95 (1.62-2.48)^d^	1.05 (0.78-1.43)
Before 11 AM	2.65 (1.96-3.57)	1.32 (0.86-2.02)
Before noon	3.51 (2.42-5.1)	1.83 (1.16-3.41)
Before 1 PM	4.54 (2.94-7)	2.68 (1.44-4.96)
Before 2 PM	5.71 (3.53-9.25)	4.06 (2.2-8.9)
Before 3 PM	6.96 (4.15-11.7)	6.14 (2.9-12.81)
Before 4 PM	8.22 (4.78-14.15)	9.01 (4.41-21.43)
Before 5 PM	9.36 (5.37-16.33)	12.44 (5.24-27.36)
Before 6 PM	10.26 (5.88-17.95)	15.65 (7.34-37.64)
Before 7 PM	10.79 (6.23-18.75)	17.40 (7.7-37.94)
Before 8 PM	10.88 (6.38-18.6)	16.57 (7.92-38.37)
Before 9 PM	10.47 (6.28-17.52)	13.11 (6.05-27.03)
Before 10 PM	9.60 (5.91-15.68)	8.35 (4.28-18.27)
Before 11 PM	8.36 (5.47-13.96)	4.16 (2.02-7.98)

^a^Mean moderate-to-vigorous physical activity (MVPA) for nonsports participators=24.45 min.

^b^Mean MVPA for sports participators=40.81 minutes.

^c^N/A: not applicable.

^d^Significant differences in odds ratios between groups based on nonoverlapping CIs.

**Table 5:** Moderating effect of school day on odds ratio estimates of time of average moderate-to-vigorous physical activity accumulation.

Hour	Odds ratio estimates (95% CI)	
	Nonschool day^a^ (1036 days)	School day^b^ (862 days)
Before 8 AM (reference)	N/A^c^	N/A
Before 9 AM	1.55 (1.63-1.77)^d^	0.99 (0.87-1.12)
Before 10 AM	2.34 (1.82-3)^d^	1.1 (0.86-1.4)
Before 11 AM	3.41 (2.4-4.83)^d^	1.34 (0.95-1.88)
Before noon	4.79 (3.08-7.39)^d^	1.76 (1.15-2.68)
Before 1 PM	6.48 (3.87-10.73)	2.43 (1.47-3.95)
Before 2 PM	8.43 (4.73-14.75)	3.44 (1.97-5.89)
Before 3 PM	10.50 (5.63-19.17)	4.88 (2.67-8.67)
Before 4 PM	12.52 (6.5-23.48)	6.77 (3.59-12.31)
Before 5 PM	14.25 (7.25-27.09)	9.00 (4.67-16.51)
Before 6 PM	15.44 (7.8-29.37)	11.16 (5.75-20.43)
Before 7 PM	15.91 (8.06-29.9)	12.65 (6.53-22.82)
Before 8 PM	15.54 (7.99-28.56)	12.78 (6.66-22.52)
Before 9 PM	14.37 (7.54-25.59)	11.26 (5.95-19.25)
Before 10 PM	12.54 (6.76-21.54)	8.44 (5.53-13.99)
Before 11 PM	10.32 (5.7-17.08)	5.27 (2.85-8.5)

^a^Mean moderate-to-vigorous physical activity (MVPA) on nonschool days=28.49 min.

^b^Mean MVPA on school days=33.82 min.

^c^N/A: not applicable.

^d^Significant differences in ORs between groups based on nonoverlapping CIs.

### Aim 3

**Figure 6:**
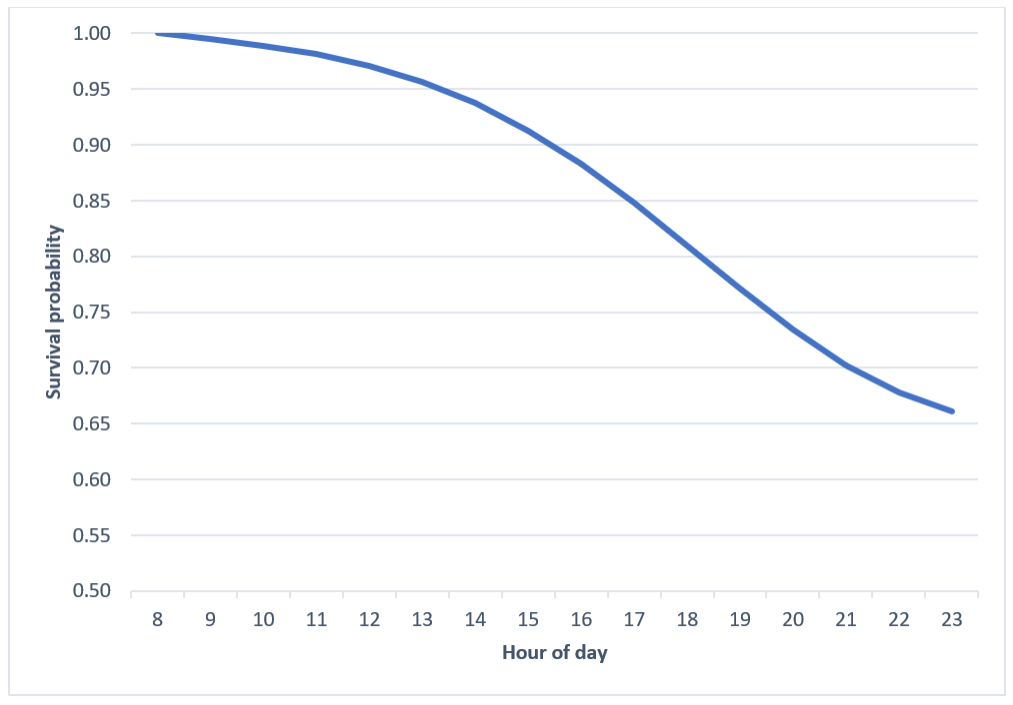
Survival probabilities of obtaining average moderate-to-vigorous physical activity before each hour of day.

On average, hazard probabilities indicated that adolescents were most likely to instantaneously meet their average MVPA between 5 PM and 8 PM (Table 6). OR estimates and hazard probabilities indicate slightly different times owing to their mathematical computation. The most likely times offered by the OR estimates and hazard probabilities generally overlap and are conceptually congruent. Survival probabilities demonstrate that after 8 PM, adolescents had a 73% chance of not meeting their own MVPA average (Table 6 and Figure 6). The sharp decline (−12%) in survival probabilities between 5 PM and 8 PM indicates that adolescents’ risk of not meeting their average MVPA drastically reduced during this time compared with the windows of time before and after this period. Intervention decision points should therefore be prioritized during this period (5 PM to 8 PM).

On the basis of the criteria set in place for generating decision points for moderators, sex was the only moderator that upheld both criteria. Although men were significantly more likely to meet their average MVPA during the 8 AM to noon time, there are essentially no differences in the hazard and survival probabilities of MVPA accumulation during this window. Nonetheless, sex differences in the OR estimates at these times indicate that the timing of activity may differ across sexes. Overall, male adolescents might benefit from additional digital support for exercise during the morning hours compared with females.

**Table 6:** Hazard and survival probabilities of time of average moderate-to-vigorous physical activity accumulation.

Hour	Hazard probability	Survival probability
Before 8 AM	0.00	1.00
Before 9 AM	0.01	1.00
Before 10 AM	0.01	0.99
Before 11 AM	0.01	0.98
Before noon	0.01	0.97
Before 1 PM	0.02	0.96
Before 2 PM	0.03	0.94
Before 3 PM	0.03	0.91
Before 4 PM	0.04	0.88
Before 5 PM	0.05	0.85
Before 6 PM	0.05	0.81
Before 7 PM	0.05	0.77
Before 8 PM	0.04	0.73
Before 9 PM	0.04	0.70
Before 10 PM	0.03	0.68
Before 11 PM	0.00	0.66

## Discussion

### Principal Findings

The purpose of this study was to explore the timing of exercise for adolescents, identify correlates of physical activity that moderate the timing of exercise, and generate decision points for digital physical activity interventions. No other studies were found that explored the time of day when adolescents meet their typical levels of MVPA or the time of day when adolescents generally exercise. The pattern of ORs, hazard probabilities, and survival probabilities indicating typical MVPA attainment in the late afternoon and early evening (5 PM to 8 PM) coincides with adolescents’ daily schedule. Generally, adolescents’ ability to exercise is constrained by their school attendance, designating early-to-mid afternoon as the earliest convenience for adolescents to meaningfully accrue MVPA [46]. Furthermore, the decline in ORs, as well as hazard probabilities after 8 PM can be explained by adolescents’ needs to attend to other important routines, such as eating dinner, completing homework, and preparing for the next day [47]. Therefore, adolescents may be less likely to engage with digital health intervention options outside of this window of time, and digital support to encourage exercise during these times could be wasteful. This persistent inopportune support is likely to lead to intervention failure and decreased user engagement, a continual challenge in the digital health literature [14-16,48]. These findings suggest that just-in-time support during this window could be most helpful for adolescents and lead to positive engagement with digital support for exercise.

These findings show that males have significantly higher odds of obtaining their average MVPA in the morning compared with females. These findings highlight that daily exercise patterns may vary by sex. Furthermore, these results suggest that male adolescents may benefit from exercise prompts in the morning *and* in the afternoon, whereas female adolescents might only benefit from exercise prompts later in the day. In other words, although it could be wasteful to prompt female adolescents to exercise in the morning, supplementary digital support for exercise in the morning (eg, 8 AM to noon) could be helpful for male adolescents. Although sending digital support for exercise in the morning to males could be conceived as wasteful, given that they are in school, research demonstrates that male adolescents obtain more exercise than female adolescents in school, including when leisurely on school grounds and also when at recess and gym class [49]. Therefore, digital support for male teenagers during this time may prove beneficial.

There were no significant differences in the timing of the attainment of typical MVPA across groups of different weight statuses. Although previous research has indicated that youths with overweight and obesity exercise less, this study did not find substantial differences in MVPA levels across weight statuses (Table 3) [25,28]. The absence of MVPA differences across groups of varying weight statues probably contributed to the lack of timing differences across these groups. In this case, BMI may not affect the actual timing of exercise. Surprisingly, nonsports participants had significantly higher odds of obtaining their typical MVPA in the morning compared with sports participators. Consistent with previous research, sports participants in this study displayed a higher mean MVPA (Table 4) [50]. Therefore, it should take longer for sports participants to accumulate their typical levels of MVPA compared with nonsports participants. This relationship between higher MVPA averages and later timing of typical MVPA attainment may explain why these results contradict the hypothesis. In addition, this finding reflects adolescents’ typical sports practice and game schedule such that sports participants may be engaging in MVPA later in the day after school practices or evening games.

There were higher odds of MVPA attainment in the morning on nonschool days than on school days, which may be indicative of the lack of constraints that prevent exercise on school days, especially considering that there were similar levels of mean MVPA across school vs nonschool days (Table 5). In other words, there may be more opportunities for adolescents to exercise in the morning on nonschool days, which might modify the timing of support on those days [30].

On the basis of this study, decision points for JITAIs promoting exercise could occur between the 5 PM and 8 PM time frame and between 8 AM and noon for male teenagers, as indicated by ORs, hazard probabilities, and survival probabilities. This period appears to overlap with adolescent exercise patterns and could serve as an optimal starting place for novice exercisers to accrue MVPA. In addition, on days when youths have met their typical MVPA by this window, this period could serve as an opportunity to make exercise gains. It should be added that survival probabilities indicated that youths are still 73% unlikely to obtain their typical MVPA after 8 PM. There were more days when adolescents did not meet their average than the days they did. This finding indicates that encouraging adolescents to consistently meet their own MVPA average would constitute a meaningful shift in MVPA attainment and a consequent increase in their typical MVPA levels, which could arrest the decline in MVPA observed during adolescence.

### Limitations and Future Directions

To determine when users engaged in exercise, this study investigated the time of day when adolescents met their mean MVPA. Some users probably do not accrue MVPA in extensive, continuous bouts of time; rather, they likely obtain MVPA over intermittent spans of time throughout the day [46]. For instance, a person may gain some MVPA walking to school in the morning, in gym class, and after school in sports practice. Therefore, users may engage in exercise multiple times throughout the day, and this study’s conceptualization of physical activity timing does not capture this pattern. Furthermore, the timing of obtaining a typical MVPA is likely to be earlier for individuals who typically accrue little-to-no MVPA compared with those who typically accrue more MVPA. Given that these results are averages across individuals, it is also important to acknowledge the potential inability to generalize this average physical activity timing profile to other samples and populations. In addition, interventions based on these results need to consider individual variability in physical activity timing based on their unique activity patterns. In other words, the decision points to improve exercise might differ between sedentary adolescents and active adolescents. For example, because sedentary adolescents might need more prompts to exercise or might have already obtained their typical levels of MVPA earlier in the day, decision points for a sedentary adolescent could occur more frequently, such as after prolonged bouts of sedentary time, whereas a more active adolescent might benefit from decision points at the times when they are usually physically active.

This study included participants recruited from different seasons of the year (eg, winter and summer), and although season may affect the timing of MVPA, by distributing data collection throughout the year, our findings are more generalizable than if they were taken from a single season. In this study, weekend days during the school year and weekdays during the summer were both classified as nonschool days, given that the lack of school during both these types of days could similarly affect the timing of activity. However, this study did not evaluate the seasonal effect of physical activity timing, which is a limitation of the study. In addition, work hours or other contexts that would constrain an adolescent’s ability to engage in MVPA were not assessed in the study. Future research should consider how these contexts would suppress one’s ability to exercise and ultimately affect their decision points.

Each of the moderators in aim 2 was analyzed independently of the others. In reality, these variables are not mutually exclusive and interact, such that there could have been unlimited interactions between these variables that moderated the most likely time of exercise. Future research should consider a more nuanced examination of how these moderators in tandem influence the timing of exercise to better optimize decision points for physical activity JITAIs across multiple contexts. Another limitation of the study is that it did not seek to determine which moderator of timing would be the most useful for adjusting decision points. Therefore, future research should investigate the experimental effects of tailoring decision points via different situational and contextual factors on improvements in physical activity.

Furthermore, it is possible that other variables might moderate the timing of exercise, including one’s built environment characteristics (eg, neighborhood walkability and access to recreational activities; [51-53]). For example, youths who actively transport (eg, biking and walking) themselves to and from school might engage in more MVPA during these windows. Moreover, the moderators evaluated in this study are mostly participant-level factors (except for the day of week). It is likely that time-varying or within-person factors also moderate typical MVPA attainment. Ultimately, this study demonstrates that it is possible to investigate how important correlates of physical activity moderate the timing of exercise. Future research should explore how additional variables, including time-varying factors, influence the timing of exercise. Such an approach would help identify dynamic receptive states to develop a truly just-in-time intervention that adapts to an individual’s changing internal and contextual state.

A key element of JITAI research and implementation is the need to identify *states of vulnerability* or *states of receptivity* for users [16,54]. A *state of vulnerability* is a dynamic state in which an individual is likely to exhibit health-compromising behaviors, whereas a *state of receptivity* is a dynamic state in which an individual is open to performing health-promoting behaviors, is likely to be responsive to health promoting cues, or is likely to be engaged with intervention options [16,54]. Decision points should, therefore, overlap with these states to deliver intervention options at critical windows of opportunity [16,54]. As stated previously, the empirically identified times found in this study represent periods when these participants were more likely to have already accumulated their typical MVPA but do not necessarily reflect periods when users are *receptive* to digital support. In other words, this study determined the time of day when adolescents are typically available for obtaining physical activity. However, this 5 PM to 8 PM availability might not completely overlap when adolescents are most responsive to digital health-promoting cues or likely to be engaged with digital intervention options. Therefore, additional research is required to determine when adolescents may benefit the most from digital support, such as investigating times of day when adolescents are most likely to exercise *and* concurrently engage with a digital health intervention.

Developing decision points for JITAIs by investigating the timing of exercise is a direct answer to calls in the research literature to model and incorporate microtemporal dynamics of health determinants or the study of behavioral phenomena in small timescales, within health behavior science [16,32,55]. Expansion of health behavior research to the microtimescale may elucidate the *temporal specificity* of health behaviors such as physical activity [16,55]. In addition, because the microtemporal study of physical activity can be continuously monitored and passively detected, JITAIs can therefore optimize and adapt interventions for each individual user more readily than in-person and static digital interventions. Most importantly, with these temporally dense data sets, researchers can statistically uncover the unique temporal patterns for each participant, given the immense quantity of observations [53,56]. It is conceivable that activity patterns may be highly idiosyncratic depending on the temporal, contextual, and psychosocial processes involved. However, with these idiographic data, automated JITAIs can be tailored to match the temporal specificity of each individual user’s microtemporal physical activity patterns. Future research exploring the timing of exercise and decision points generally should, therefore, consider employing idiographic methods, such as precision medicine approaches, to enhance decision points for each user [57,58].

Ultimately, the notion that improved timing of support for digital physical activity interventions will lead to improvements in physical activity requires experimental testing. This study demonstrates that decision points can be empirically defined and that the timing of exercise may differ among groups of people but did not evaluate the impact of tailoring these decision points on adolescent physical activity. To address this gap, it should be empirically tested if sending digital support at empirically identified moments leads to more exercise compared with digital support at interventionist-defined or user-defined times. Major areas for future research include investigating the impact of sending digital support at the empirically identified window (5 PM to 8 PM) on physical activity, evaluating adolescent receptivity to digital support during this time as well as generating and tailoring idiographic decision points based on an individual’s unique physical activity patterns.

## Abbreviations

CDC: Center for Disease Control and Prevention
JITAI: just-in-time adaptive intervention
KU: University of Kansas
MVPA: moderate-to-vigorous physical activity
OR: odds ratio
